# Depression, Is It Treatable in Adults Utilising Dietary Interventions? A Systematic Review of Randomised Controlled Trials

**DOI:** 10.3390/nu14071398

**Published:** 2022-03-27

**Authors:** Simone O’Neill, Michelle Minehan, Catherine R. Knight-Agarwal, Murray Turner

**Affiliations:** Faculty of Health, University of Canberra, Bruce, ACT 2617, Australia; simone.oneill@canberra.edu.au (S.O.); cathy.knight-agarwal@canberra.edu.au (C.R.K.-A.); murray.turner@canberra.edu.au (M.T.)

**Keywords:** dietary intervention, whole diet, whole food, depression

## Abstract

This systematic literature review examined whole food or whole diet interventions to treat depression. The inclusion criteria encompassed adults, depression, a recognized depression scale and a whole food or diet intervention. APA PsychINFO, CINAHL, the Cochrance Central Register of Controlled Trails, MEDLINE and Scopus were searched for original research addressing diet as a treatment for depression in adult populations. The quality of the study was assessed using the Academy of Nutrition and Dietetics Quality Criteria Checklist. Seven studies; with 49,156 participants; met the eligibility criteria. All these studies found positive outcomes with depression levels decreasing after dietary intervention. The calculated effect size varied from small (Cohen’s *d* = 0.32) to very large (Cohen’s *d* = 1.82). The inconsistent nature of the studies limited the synthesis of the data. Recommendations are provided to enhance future study design and measurement outcomes. Overall, the findings show a positive result for diets that promote an increased intake of fresh produce, wholegrains, low-fat dairy and lean protein sources, while also decreasing the intake of processed and high-fat foods. No funding was provided for this review. The protocol for this review is registered with PROSPERO (CRD42020210426).

## 1. Introduction

Depression, a principal basis of global disability, is a key factor in the burden of worldwide disease with an estimated 264 million individuals suffering worldwide [1]. Economically this impacts governments through increased social security payments and higher demands on healthcare systems, whilst also decreasing income through taxes [2]. Depression has consequences at the individual and community levels, such as reduced employment, the breakdown of relationships and potentially suicide [3,4]. Additionally, depression is known to have detrimental effects on physical health, further compounding the potential issues that arise from a depressive episode [1].

The pathophysiology of depression is not completely understood, but biological and psychosocial factors influence the development of depression with interactions between genetics and the environment possibly involved [5]. Currently, depression is treated using a combination of pharmacological and psychotherapy methods [5] with varying degrees of effectiveness, particularly in regard to chronic depression [6,7,8]. Dietary modification may offer a possible alternative or concurrent treatment for depression, but there is a need for clarity regarding the efficacy of dietary intervention [9].

In a number of meta-analyses of epidemiological studies, diet quality has been found to be inversely related to depression [10,11]. Lassale et al. [10] concluded that the intake of a Mediterranean diet led to a lower incidence of depression. Further support for links between decreased depression levels and high-quality diets is provided by Molendijk et al. [11] and Wu et al. [12]. Lassale et al. [10] also found a low inflammatory diet decreased the incidence of depression in women. This suggests that diet should be considered as a modifiable risk factor for depression [10].

Currently there are a number of hypothesised mechanisms for the role of diet in depression. Increased inflammation levels are thought to influence various physiological functions that are related to depressive disorders [13]. Elevated levels of oxidative stress markers have also been associated with increased levels of depression [13]. Consumption of a high-quality diet with anti-inflammatory properties and an increased supply of antioxidants may reduce systemic inflammation and oxidative stress, thereby potentially decreasing depressive symptomology. Studies have also found the gut microbiome may be implicated in increasing the risk of depression through increased inflammatory cytokines and other metabolites that are released by the microbiota [14]. Therefore, diets high in fibre and probiotics that support healthy microbiota may reduce the incidence of depression. Other physiological effects of depression include an increase in cortisol production, leading to the disruption of the hypothalamic-pituitary-adrenal (HPA) axis, decreased levels of brain-derived neurotrophic factor (BDNF) and impaired mitochondrial ATP production, possibly causing diminished neurogenesis and dysfunctional neuronal plasticity [13]. Studies have found that diets rich in vitamins, polyphenol compounds and omega-3 fatty acids support these physiological pathways and functions [13]. For a more in-depth discussion of these mechanisms refer to Marx et al. [13] and Jang et al. [14].

As humans do not consume micronutrients in isolation, it is more meaningful to consider the effects of whole foods and whole dietary patterns in relation to depression [15]. A small number of reviews adopting a whole food/whole diet lens have concluded that dietary interventions offer a potential treatment for depression [10,16,17].

This review provides different but complementary findings to a recent meta-analysis that included multi-component interventions, dietary counselling interventions without measuring if dietary change actually occurred and participants with comorbidities [18]. Firth et al. [18] considered the impact of dietary intervention on mental well-being and concluded that there is support for dietary interventions to be used to aid in reducing depression. However, it was also noted that further research is required to better identify the specific components of diet required to improve mood and mental health. This paper differs from other reviews in that it assessed whole-food/diet interventions that had no intention of causing weight loss as an outcome, in healthy individuals without co-morbidities, such as type 2 diabetes mellitus. A tight focus was kept on studies that directly measured the dietary change caused by the intervention. Interventions that were based on counselling without a clear, measurable outcome were not considered eligible.

This systematic review aims to understand the effectiveness of whole food or whole diet dietary interventions to support the treatment of depression in healthy adults.

## 2. Materials and Methods

Details of the protocol for this SR were registered on PROSPERO and can be accessed at https://www.crd.york.ac.uk/PROSPERO/display_record.php?ID=CRD42020210426 (accessed on 30 December 2021)—Registration number: CRD42020210426.

### 2.1. Data Source/Literature Search

The reporting of this systematic review was guided by the standards of the Preferred Reporting Items for Systematic Reviews and Meta-Analyses (PRISMA) Checklist [19]. APA PsycINFO, CINAHL, Cochrane Library (Database of Systematic Reviews and Central Register of Controlled Trials), MEDLINE and Scopus were searched for articles published between January 2000 and September 2021. A pragmatic decision was made to limit the search to post-2000. The food supply has changed significantly in the last 20 years with an increasing availability of processed foods [20]. It was felt that examining research post-2000 would capture dietary interventions made within a modern food supply and hence reflect the most current understanding of the relationship between dietary intake and depression. It is acknowledged that this is a potential limitation of the paper. The following search terms in combination with medical subject headings (MeSH) were used: Depression AND (Treatment Efficacy OR Treatment Effectiveness OR Intervention Efficacy OR Intervention Effectiveness) AND (Diet OR Food OR Nutrition OR Dietary Intake OR Food Intake OR Nutrition Intake). The full search terms are provided in Supplement S1. Additional articles were identified from the references of published studies, through handsearching of the literature and additional consultation with experienced authors in the field. Two reviewers independently evaluated publications for inclusion, based on their titles and abstracts. Full texts were then retrieved for those articles deemed eligible and considered for inclusion independently by both reviewers. A third reviewer was used to mediate any inconsistencies.

### 2.2. Eligibility Criteria

#### 2.2.1. Type of Participants

Studies including adults (18 years or older) were considered. Studies were included if the population was suffering from a medically diagnosed major depressive disorder or self-diagnosed depression or healthy individuals where depressive symptomology was measured by a recognized scale as an outcome of the study. Disorders such as antenatal and postnatal depression, bipolar disorder and seasonal affective disorder were excluded. Studies where participants had common chronic health conditions such as diabetes were included provided the intervention focused on depression and not the secondary disease state. Other conditions with known correlations to levels of depression, such as cancer, were excluded. Studies that utilised an intervention specifically designed to induce weight loss were excluded due to the confounding nature of weight loss on levels of depression [21].

#### 2.2.2. Type of Intervention

Whole food or whole diet interventions were included. A whole food intervention involved the consumption of a minimally processed, unfortified food item that is easily accessible in the Western food chain, for example, orange juice. A whole diet intervention adjusted or monitored the entire dietary intake. Whole food/whole diet interventions will inevitably influence the delivery of a variety of potentially active ingredients. We have included studies where a variety of food components have been manipulated provided unfortified food/s was used as the intervention rather than fortified products or supplements. Studies examining supplements, such as fish oil capsules or single micronutrients, were excluded, as were mixed method studies such as those that examined the combined effect of diet and exercise.

#### 2.2.3. Type of Studies

Only randomised controlled trials were considered, all other study designs were excluded.

#### 2.2.4. Type of Outcomes

This review considered the outcome on levels of depression as measured by a validated tool such as the Beck Depression Inventory [22,23]. Studies that relied on measures of quality of life such as the Health-Related Quality of Life (HRQoL) were not included as this is not a direct measure of depression.

### 2.3. Risk of Bias

The risk of bias was assessed using the Academy of Nutrition and Dietetics Quality Criteria Checklist (ANDQCC): Primary Research Tool [24]. The tool assesses the following factors: Q1, clearly stated research question; Q2, unbiased selection of participants; Q3, comparable study groups; Q4, description of withdrawals; Q5, blinding procedure; Q6, description of interventions; Q7, clearly defined outcomes and valid and reliable measurements; Q8, appropriate statistical analysis; Q9, results and conclusion align; and Q10, unlikely bias due to funding. The study’s quality was classed as positive if the majority of these criteria were met with definite positive outcomes for criteria 2, 3, 6 and 7 as well as one other validity criteria question, neutral if criteria points 2, 3, 6 and 7 did not score a ‘yes’, or negative if more than six of the validity criteria questions were answered with a ‘no’. Two reviewers independently assessed all included studies, with a third reviewer consulted to mediate any inconsistencies. The studies were assessed on 10 criteria addressing the validity of the studies including bias and this resulted in an overall rating of positive, neutral or negative.

### 2.4. Data Extraction

Data were extracted from included papers based on their aims, location, sample size, participant characteristics, follow-up period, intervention, control group protocol and outcomes/results. In accordance with recommendations outlining the benefits of considering effect size [25], Cohen’s *d* was calculated for the five studies that provided means and standard errors or standard deviations. Where standard error was reported, this was converted to the standard deviation by multiplying the standard error by the square root of the sample size. All calculations were based on the difference between the mean at baseline and post treatment, with effect size calculated for all possible studies to allow for consistency in comparison. No meta-analysis was undertaken as the lack of consistency in the scales used to measure depression precluded any direct comparison of the results.

## 3. Results

### 3.1. Study Selection

**Figure 1:**
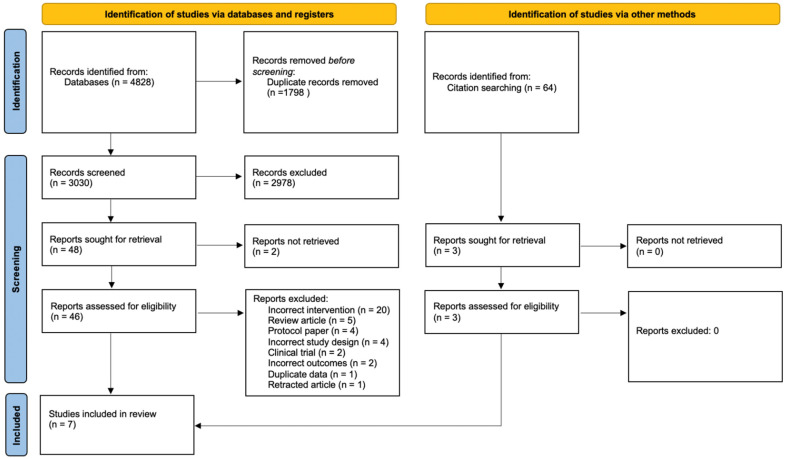
PRISMA flow diagram of studies included in the review.

The initial search resulted in a total of 3030 studies. Of these 2978 were eliminated through the initial screening process. Of the remaining 48 studies, 41 were eliminated for the following reasons: incorrect intervention (*n* = 20), review article (*n* = 5), protocol paper (*n* = 4), incorrect study design (e.g., cohort study, *n* = 4), clinical trial (*n* = 2), unable to access paper (*n* = 2), wrong outcomes assessed (*n* = 2), duplicated data (*n* = 1) or retracted article (*n* = 1). Three additional studies were identified through bibliographic searches of relevant literature. Seven studies were included in the review. Figure 1 presents the outcomes of the study selection process.

### 3.2. Quality Assessment

The quality assessment outcomes were that four studies received a positive rating and three a neutral rating against the ANDQCC (Table 1).

### 3.3. Study Characteristics

#### 3.3.1. Location and Sample Size

Three of the studies were undertaken in Australia and two in the USA, with the remaining trials being conducted in Korea and the UK. The seven studies examined included a total of 49,156 participants with sample sizes ranging between 25 and 48,835 individuals.

#### 3.3.2. Population Characteristics

All studies targeted adult populations (e.g., ≥18 years) except one [27] which included participants aged from 17 to 35 years. Of the remaining six studies, two considered adults across the lifespan [28,29,33], two young adults (e.g., 18–30 years) [31,32] and one examined older participants (50–79 years) [26]. Three studies identified existing depressive symptomology, either self or medically diagnosed [27,28,32], and one study focused on a diagnosis of hypertension [29]. Three studies targeted populations with poor diet quality [26,27,28]. The majority of the studies included males and females; however, two studies [26,31] involved exclusively female participants and another did not specify a gender breakdown [30]. Additional details are found in Table 2.

#### 3.3.3. Study Aims and Primary Outcomes

Four of the studies utilised a whole diet intervention [26,27,28,31]. Of these, two focused solely on depression as the primary outcome [27,28]. McMillan et al. [31] also considered cognitive function, while Assaf et al. [26] also measured the effects of the intervention on health-related quality of life, self-reported health, cognitive function and sleep quality. The remaining studies used whole food-based interventions to increase the intake of a targeted food chemical [29,30,32] e.g., polyphenols. These studies measured mental health [29], depression [29,30,32], anxiety [29,30], mood [29,30] and gut microbiome changes [32].

### 3.4. Intervention Description

#### 3.4.1. Session Details and Follow-Up Duration

The included studies ranged in length from 10 days to one year, with the majority of interventions proceeding for between two and three months (*n* = 4) [27,28,29,32]. Face to face contact varied between the studies, with the most common model utilising three meetings throughout the intervention (*n* = 2) [29,32]. The most intense studies connected with the participants for seven sessions (1 h duration) across the intervention [28] or were in daily contact during the two intervention phases [30]. The longest running study conducted 18 group sessions over the course of the year [26]. The minimum contact was an assessment conducted at baseline and upon the completion of the intervention [31].

#### 3.4.2. Intervention Style and Programme Components

All interventions were dietary, with studies focusing on whole diet including the Mediterranean diet (*n* = 2), dietary guidance to reduce fat intake to 20% of caloric intake and to increase whole grain and fruit and vegetable intake (*n* = 1) and a diet based on the Australian Guide to Healthy Eating (*n* = 1) or on an individual phytochemical (*n* = 3). Studies were conducted through face-to-face group sessions (*n* = 5) or individual contact (*n* = 3) with face-to-face meetings, phone calls and electronic web-based methods utilised. In two studies, the control group was directed to maintain their normal diet [27,31]; one study had a control arm specifically designed to match the social interactions without the dietary intervention [28], while the other five studies compared the mean difference in change from baseline to the conclusion of the study between the intervention and control group. Dietary compliance was assessed through 24-h recall [32], food frequency questionnaires [26,32], food diaries [29,31], provision of all food consumed [30], daily written verification of compliance [30] or dietary questionnaires [28]. Five studies specifically provided detail on final compliance [27,29,31,32] with McMillan et al. [31] reporting that 93% of meals and 95% of snacks in the dietary change group meet the criteria for the diet implemented for the study and Park et al. [32] and Kontogianni et al. [29] outlining changes in energy, protein, fat and various micronutrients or shifts in intake of specific food groups. Assaf et al. [26] utilised food frequency questionnaires to assess compliance. Another study measured changes in fruit and vegetable intake via spectrophotometer scores [27]. In one study, participants were specifically requested to maintain their existing physical activity levels [32]. The majority of studies had a dietitian or nutritionist guiding advice (*n* = 6). Studies also utilised psychologists (*n* = 2), pharmacists (*n* = 1), a biostatistician (*n* = 1), nurses (*n* = 1), a physiotherapist (*n* = 1) and personnel trained to support implementation of the intervention (*n* = 2). Two studies did not specify the qualifications of the investigators involved [29,31].

#### 3.4.3. Depression Outcome Measures

A wide variety of depression scales were used in the assessment of depressive symptomology. Additional details are included in Table 3.

The studies matched intervention and control groups on a range of baseline characteristics, ensuring that the groups were not significantly different on a number of factors, including age, gender and a measure of body composition, either BMI, waist circumference or weight. Other characteristics considered included education levels (*n* = 4), measures of socioeconomic status (*n* = 2) and lifestyle factors including physical activity (*n* = 2), smoking (*n* = 4) and binge drinking (*n* = 1). Health related characteristics were also considered including comorbidities such as cholesterol levels (*n* = 1), blood pressure (*n* = 2) and various biochemical measures (*n* = 1). Additionally, three studies considered baseline depression and anxiety levels.

### 3.5. Results for Depression

At the conclusion of the intervention, all studies revealed a decrease in depressive symptomology. For the five studies that Cohen’s d could be calculated, the effect size ranged from Cohen’s *d* = 0.32 to 1.82. The effect size was classified as trivial (Cohen’s *d* ≤ 0.2), small (>0.2), moderate (>0.5), large (>0.8) or very large (>1.3) [25]. Of the five studies, three studies [30,31,32] showed a small effect, one study [27] showed a small and medium effect depending on the depression scale used and one study [28] showed a large effect. The study examining flavonoid intake resulted in decreased depression scores (FR *p* < 0.0001 and FL *p* = 0.001) for both the rich and low interventions with a greater effect size found for the high intervention (Cohen’s *d* FL = 0.31, Cohen’s *d* FR = 0.37) suggesting a dose-related effect for flavonoids in relation to depressive symptomology.

### 3.6. Quality Rating

The studies received a positive quality rating (*n* = 4) or a neutral rating (*n* = 3). The common issues with those studies that received a lower quality rating included concerns regarding target population and subject selection, statistical analysis and funding sources.

## 4. Discussion

This systematic review of RCTs assessed the efficacy of dietary interventions (whole food and whole diet approaches) on depressive symptomology. All included studies showed a reduction in depression measures from baseline to conclusion. The study undertaken by Lindseth et al. [30] provides additional evidence that consuming a diet low in tryptophan may potentially increase depression symptomology. Previous examination of tryptophan intake and urinary outputs from the kynurenine metabolic pathway in an elderly cohort found depression was linked with a decreased intake of tryptophan and an increased output of metabolites associated with the kynurenine metabolic pathway [34]. This study [30] had a clearly defined intervention where the adjustment of dietary tryptophan was based on 5 mg/kg body weight for the low level intervention and 10 mg/kg body weight at the high level intervention. The participants were provided all food required to ensure dietary compliance. However, a major drawback in the study was the short time frame, 2 weeks, for both interventions. The two studies [29,32] that assessed foods high in polyphenols and flavonoids provide further support to previous epidemiological evidence that intake of these phytochemicals can alleviate symptoms of depression through a proposed mechanism relating to the antioxidant and anti-inflammatory nature of these phytochemicals [35,36]. A limitation of these studies is that it is difficult to ensure the consistency of the phytochemical provided in food as these vary due to factors such as growing climate, ripeness at harvesting and food processing [37]. This also has implications for implementation as a potential treatment option in the future. Each of the phytochemicals discussed here are found abundantly within a Mediterranean diet, as is tryptophan [35,36,38]. This provides additional support for the examination of this diet model and similar models in the remaining three studies [26,27,28] that showed positive outcomes. Furthermore, the literature reports a strong correlation between adherence to a Mediterranean diet or a similar style of eating and decreased risk of depression [10].

The final study [31], utilizing a 10-day Mediterranean dietary intervention, found a small effect (Cohen’s *d* = 0.41) in depression levels. The short duration may potentially account for this outcome, which supports the need for greater consideration regarding intervention duration as with more time a larger effect may have become apparent. The study undertaken by Park et al. [32] showed a positive outcome regarding depression for both the flavonoid rich (Cohen’s *d* = 0.37) and flavonoid low (Cohen’s *d* = 0.32) diets for depression, but with a slightly greater effect size for the flavonoid rich intervention. Again, each intervention phase was of very short duration which may have affected the outcomes, with more time and longer follow-up periods required to ensure the validity of the conclusions.

The wide variety of measures, such as the BDI-II, DASS-21 and the CES-D, used to assess changes in depression symptomology makes comparison between the studies difficult, thus limiting the synthesis of the available data and therefore clear recommendations for the use of dietary interventions as a treatment model for depression. All interventions, except two which did not provide details of delivery [29,31], were administered by relevant health professionals as is supported by previous evidence showing improved outcomes when dietary interventions are delivered by qualified clinicians [39,40] such as dietitians.

This systematic review assessed the capacity for whole food/diet interventions to improve depressive symptoms. A tight focus was kept on studies that directly measured dietary change. This review provides different but complementary findings to a recent meta-analysis that included multi-component interventions and dietary counselling interventions without measuring if dietary change actually occurred and participants with comorbidities [18].

### 4.1. Strengths and Limitations

The strength of this review is that it considered only randomised control trials that met very specific criteria that were determined prior to the commencement of database searches. Use of the PRISMA process further adds validity to the selection processes undertaken during the identification of studies to be included.

A limitation frequently found in nutrition studies, and a limitation for the current review, is accurate measurement of dietary intake [15,41]. To strengthen the validity of the self-reported data, in which dietary intake is usually under-reported [42], additional confirmation through biomarkers may be valuable [42,43]. The RCTs in this review used a variety of methodologies to ensure dietary compliance and assess dietary intake including providing all food consumed [30], questionnaires designed to assess dietary intake and quality [28,44], food diaries [28,29], and 24 h recalls [32]. Three of the studies [28,29,32] reported blood lipid or other biomarkers and spectrophotometry related to dietary intake; however, no links were made between these and dietary intake or compliance in all but one study [27].

Further complexities arise when consideration is given to the differences that exist in the emphasis each individual depression scale places on various symptom domains, for example, physical effects such as impact on appetite versus mood and changes including the feeling of sadness or anxiety [22,45,46,47]. The different emphases on the various symptom domains can lead to variation in scores across multiple measures, making comparisons between results from different studies worthless [46,47,48]. Additionally, different scales were developed for varying purposes; for example, the BDI-II was developed for use as a diagnostic tool, while the CES-D was designed for use in epidemiological studies of depression [23], further complicating the impact different scales have on study outcomes. Furthermore, while HADS/MADRS are used in research settings, some researchers are critical of this and recommend they be limited to clinical use [23,48]. The combined effect of the lack of accuracy in measuring both dietary intake and mental health status may result in smaller changes being overlooked. Therefore, the study design and the selection of tools used for assessment need to be undertaken carefully with a clear understanding of their limitations.

Depression interacts with factors including gender [49], race [50], physical activity [51], sleep [52], alcohol consumption [52] and smoking [53]. Due to the effects of these factors on depression, controlling for these would increase the validity of the research. Within the studies included in this review, one study did not specify the gender breakdown [30]; five studies had a mix of genders [27,28,29,30,31,32], with each intervention group matched; and in two studies all participants were female [26,31]. Three studies considered smoking [26,28,29]. To increase the validity of future studies, clear explanations of participant characteristics needs to be provided and confounding factors of depression need to continue to be addressed with well-defined details provided on how these have been dealt with in the study framework.

The blinding of both participants and investigators is challenging in dietary intervention studies [54]. The current studies used a variety of methods to blind participants, investigators or both to group appropriation. Blinding techniques included allocation by assistants with no further involvement [31], allocation by investigators delivering the intervention but not conducting the analysis [28] and the provision of partial information about objectives to the participants [27,28]. Three studies did not include details of blinding procedures beyond stating it happened [29,30,32]. A final study did not disclose whether blinding occurred [26]. To increase the strength of future studies careful attention to protocol design with clear, detailed methodology for blinding to nutrition interventions is required.

Casacalenda et al. [7] concluded that for efficacy to be apparent in the treatment of depression through pharmacotherapy and psychotherapy, a period between three to four months was required. Intervention duration is an important consideration for study design, as positive outcomes may be reduced or the effect size too small if insufficient time is allowed. While all the studies in the current review resulted in a decrease in depressive symptomology, only two interventions were conducted for three months or longer [26,28]. The study with the briefest intervention of 10 days using a Mediterranean diet [31] showed only a small effect. This outcome conflicts with the results of the study by Jacka et al. [28] using a Mediterranean diet over a three-month period, which found a larger effect, highlighting the importance of ensuring sufficient duration. Furthermore, the broad timespans of successful interventions emphasize the current lack of understanding regarding the optimal duration required for positive outcomes to occur when using a nutrition intervention.

When interpreting the results of these studies additional consideration needs to be given to sample size. A major limitation of this review is the small sample size in six of the seven studies. Small sample sizes are underpowered with the reported effect sizes being biased and too large [55,56] leading to overstated outcomes.

### 4.2. Future Research Implications

For future investigations into the efficacy of dietary interventions as a treatment for depression, a number of recommendations are suggested. Firstly, the selection of participants needs to consider current dietary intake, providing a clear starting point. Alternatively, the use of a ‘washout’ period prior to commencement of the intervention as undertaken by Kontogianni et al. [29] also provided a clear baseline.

There are a number of considerations regarding study interventions. Firstly, interventions should be delivered by qualified professionals to maximise potential outcomes [39,40]. Appropriate professionals delivered the education in the studies within the current review and it is highly encouraged that this practice continue. However, further consideration needs to be given to intervention duration, with a variety of timespans utilised in the included studies. Current recommendations for pharmacological and psychotherapy interventions are that they continue for a period of three to four months [7]. Therefore, in future, dietary interventions to manage depression should have a minimum duration of three months. A clear measure of any dietary change is vital to attribute any effects to an intervention. Therefore, the recommendation is that protocols include measures of dietary compliance. The studies reviewed utilised a variety of compliance measures including 24-h dietary recalls, food frequency questionnaires, food diaries and diet-specific questionnaires. The most stringent control possible involves providing all food and drink for participants [30]. Additional confirmation of dietary compliance can be obtained through biochemical markers [43]. Only one utilised biochemical markers [27] and, where possible, the inclusion of validated biomarkers is recommended to strengthen the quality of future research. Furthermore, consideration needs to be given to the bidirectional influences between nutrition and mental health, such as when a depressed individual lacks the motivation to purchase and prepare quality food [57], which were not considered in any of the reviewed studies.

Finally, the choice of a relevant depression scale from the plethora available needs to be undertaken with care. Other authors have previously commented that selection is often arbitrary or based on what is customary; however, the instrument selected can have a significant influence on the outcome and on the possibility of comparisons across studies [22,23,58]. The possible impact is evident when considering the differing effect sizes for the CES-D (Cohen’s *d* = 0.48) and DASS-21 (Cohen’s *d* = 0.59) found by Francis et al. [27]. Santor et al. [45] identified five commonly used scales in treatment outcome studies: the Hamilton Rating Scale for Depression (HRSD), BDI-II, Symptom Checklist—90 (SCL-90), MADRS and the CES-D. Of these only the HRSD, the BDI-II and the CES-D are recommended for research purposes [23]. Therefore, it is recommended that one of these scales is used for future research in this area.

## 5. Conclusions

The current review provides some support for whole diet and whole food interventions as an adjunctive treatment to improve depression symptomology. The available studies are limited by factors such as duration and small sample sizes, and are inconsistent in design. However, all studies showed a reduction in scores assessing depression. This suggests that whole food and whole diet interventions should be further investigated to identify the mechanisms and durations required for improved outcomes. Further studies in wider population groups are required, as is a greater control of confounding factors and their impacts on depression, along with a greater care of selection of the depression scales used to measure outcomes.

## Figures and Tables

**Table 1:** Quality assessment of included studies.

Author and Year	Q1	Q2	Q3	Q4	Q5	Q6	Q7	Q8	Q9	Q10	Overall Rating
Assaf et al. (2016) [26]	U	Y	U	N	U	N	Y	Y	Y	Y	Neutral
Francis et al. (2019) [27]	Y	Y	Y	Y	Y	Y	Y	Y	Y	N	Positive
Jacka et al. (2017) [28]	Y	Y	Y	Y	Y	Y	Y	Y	Y	Y	Positive
Kontogianni et al. (2020) [29]	Y	Y	Y	Y	U	Y	Y	U	Y	Y	Positive
Lindseth et al. (2015) [30]	Y	U	N	U	Y	U	Y	N	U	Y	Neutral
McMillan et al. (2011) [31]	U	N	Y	Y	Y	Y	Y	N	Y	N	Neutral
Park et al. (2020) [32]	Y	Y	Y	Y	U	Y	Y	N	Y	Y	Positive

Y—Yes (criteria met); N—No (criteria not met); U—Unclear.

**Table 2:** Characteristics of Included Studies.

Reference, Country	Population, Eligibility Criteria	Sample Size	Intervention	Depression and Diet Measures	Primary Statistical Outcomes
Assaf et al. (2016); USA [26]	Women aged 50–79; Baseline fat intake 32% of total calories	**Intervention** DM, *n* = 19,541 **Comparison** CG, *n* = 29,294 given no dietary instruction	Reduced fat, healthy diet intervention. Total dietary fat 20% of energy with individual goals set based on height. Fruit and vegetables 5 servings/day. Grains 6 servings/day. Group education sessions delivered by trained nutritionists.	RAND 36-Item Health Survey Subscale; Dietary Compliance—FFQ	RAND 36 DM mean score change (−0.05) significantly greater than mean score change CG (−0.12) Mean difference (0.07 [95%CI 0.02 to 0.12; *p* = 0.009])
Francis et al. (2019); Australia [27]	Individuals aged 17–35; with a score of 7 or more on the DASS-21 and greater than 57 on the DFS with antidepressant use greater than 2 weeks if relevant	**Intervention** DC, *n* = 38 **Comparison** HD, *n* = 38; given no dietary instruction	Diet based on AGTHE and Mediterranean diet (decreased refined carbohydrate, fatty or processed meats and soft drinks). Education delivered by a qualified dietitian: face to face contact at baseline and day 21; phone contact at days 7, 14 and 3 months.	CESD-R; DASS-21; Dietary Compliance—Diet Compliance Score Questionnaire	CESD-R (DC/HD—day 21) significantly lower than HD at day 21 (F[1.75] = 7.792, *p* = 0.007, Cohen’s d = 0.65); when age, gender, physical activity and baseline BMI controlled for, signficance remained (F[1.71] = 7.091, *p* = 0.010;
Jacka et al. (2017); Australia [28]	Individuals aged >18 years that meet DSM-IV diagnostic criteria, have a score of 18+ on MADRS and a score of <75 on a dietary screening tool	**Intervention** DSG, *n* = 31 Dietary Counselling **Comparison** SSCG *n* = 25; Non-dietary counselling	Improved diet quality with recommended servings specified for 12 key food types. Seven 1-h individual dietary support sessions—weekly for first four weeks, and then fortnightly for six weeks; delivered by a clinical dietitian	MADRS; Secondary Measures—HADS and POMS; dietary compliance—ModiMedDiet via 7-day food diaries	MADRS—T(60.7) = 4.38, *p* < 0.001; Cohen’s *d* = 1.16 (95% CI −1.73, −0.59); After controlling for covariates *t*(58.7) = 4.40, *p* < 0.001; mean (±SE)
Kontogianni et al. (2020); UK [29]	Individuals aged 40–65 years with documented grade I or II hypertension	**Intervention** HPD group, *n* = 50 **Comparison** LPD group, *n* = 49 Continued with ‘washout’ diet	High antioxidant diet: 4 week ‘washout’ period with <2 fruit and vegetable portions daily plus exclusion of berries and dark chocolate. An 8 week period with consumption of 6 portions of fruit and vegetable (including a portion of berries) and 50 g of dark chocolate daily. Group education baseline and week 4. Qualifications of professionals delivering intervention not stated.	PANAS; BDI-II (21-item scale); DASS-21 (21-item scale); dietary compliance—4-day food diary at weeks 4 and 12	BDI-II;—significant between-group difference (*p* = 0.01) for depressive symptoms;—significant within-group (HPD) difference 3.4 (*p* < 0.001) for depressive symptoms; PANAS—significant within-group (HPD) for PA 2.2 (*p* = 0.03).
Lindseth et al. (2015); USA [30]	University students aged > 18 years	**Intervention** HTD, *n* = 25 **Comparison** LTD, *n* = 25 within subject, crossover, double blinded study	High tryptophan diet: 4 days of meals that met EER and US RDA (5% variance). Caffeine limited to 100 mg/d. LTD phase—5 mg/kg body weight/d of tryptophan; HTD phase—10 mg/kg body weight/day of tryptophan. All meals provided in dining room, 2 week washout period between phases. Intervention conducted by dietitian, nurse and psychologist.	Zung’s SDS; PANAS direct observation of meal consumption	Within-subject analysis found low levels of tryptophan intake associated with increased rate of depression (paired *t* = 2.2, *p* = 0.02)
McMillan et al. (2011); Australia [31]	Females aged 19–30 years	**Intervention** DC *n* = 12 **Comparison** NC *n* = 13; Continued usual daily diet	Healthy diet intervention. Increased fruit, vegetables, fatty fish, nuts, seeds, low-fat natural dairy and wholegrain cereals; excluded red meat, refined sugars and flour, pre-packaged and processed foods, caffeinated products, soft drinks and condiments.	POMS; Bond-Lader VAS; dietary compliance—daily food diary	No significant change for depression; mean (±SD); Pre 21.92 ± 7.23; Post 18.83 ± 5.44
Park et al. (2020); Korea [32]	Individuals aged between 20–30 years with a CES-D score ≥ 21	**Intervention** FR *n* = 20; FL *n* = 20 **Comparison** *n* = 40; Pre-treatment sample	High flavonoid whole food intervention. Participants continued usual exercise and diet, limited HF and high-sucrose foods, fruits, juice, tea, jams and alcohol. 30–60 min before breakfast and dinner consumed 190 mL juice that naturally provided high (FR 157.9 mg/100 g) or low (FL 28.4 mg/100 g) level of flavonoids.	CES-D; dietary compliance—24 h dietary recall and FFQ	CES-D scores decreased to <20 points. Multiple regression analysis showed CES-D in FR decreased significantly (*p* < 0.0001) compared to FL (*p* < 0.001) post intervention

DM, dietary modification; CG, control group; FFQ, Food Frequency Questionnaire; DC, Dietary Change; AGTHE, Australian Guide to Healthy Eating; HD, Habitual Diet; CESD-R, Center for Epidemiological Studies Depression Scale—Revised; DASS-21, Depression, Anxiety Stress Scale-21; BMI, Body Mass Index; DSM-IV, Diagnostic and Statistical Manual of Mental Disorders—IV; MADRS, Montgomery-Åsberg Depression Rating Scale; DSG, Dietary Support Group; SSCG, Social Support Control Group; HADS, Hospital Anxiety and Depression Scale; POMS, The Profile of Mood States; HPD, High Polyphenol Diet Group; LPD, Low Polyphenol Diet Group; PANAS, the Positive and Negative Affect Scale; BDI-II, Beck Depression Inventory-II; PA, Positive Affect; EER, Estimated Energy Requirements; US, United States; RDA, Recommended Dietary Allowance; LTD, Low Tryptophan Diet; HTD, High Tryptophan Diet; SDS, Self-Rating Depression Scale; NC, No Change; Bond-Lader VAS, Visual Analogue Scales; SD, Standard Deviation; CES-D, Center for Epidemiological Studies Depression Scale; HF, High Fat; LF, Low Fat; FR, Flavanoid Rich; FL, Flavanoid Low.

**Table 3:** Depression Measures Utilised.

Author and Year	RAND36	BDI-II	Bond and Lader VAS	CES-D/CESD-R	DASS-21	HADS	MADRS	PANAS	POMS/POMS-A	Zung’s SDS
Assaf et al. (2016) [26]	√									
Francis et al. (2019) [27]				√	X				X	
Jacka et al. (2017) [28]						X	√		X	
Kontogianni et al. (2020) [29]		√			X+			X		
Lindseth et al. (2015) [30]								X		√
McMillan et al. (2011) [31]			√						√	
Park et al. (2020) [32]				√						

√—Primary Measure of Depression; X—Secondary Measure of Depression; +—Introduced 9 Months into Study.

## Supplementary Materials

The following are available online at https://www.mdpi.com/article/10.3390/nu14071398/s1, Supplement S1: Search strategy.
