# Supplementary material for: Depression, Is It Treatable in Adults Utilising Dietary Interventions? A Systematic Review of Randomised Controlled Trials
Source: Nutrients. 2022 Mar 27;14(7):1398. doi: 10.3390/nu14071398 (PMC9003461; doi:10.3390/nu14071398)
Supplement: Supplementary file 1 [file nutrients-14-01398-s001.zip › nutrients-1561317-supplementary.pdf]

Supplementary Material

Supplement 1 – Search strategy

Five databases were searched on 2 September 2021 to identify relevant studies (APA PsycINFO (EBSCOhost), CINAHL (EBSCOhost), Cochrane Library (DSR and CENTRAL), Medline (EBSCOhost), and Scopus. A limiter was applied to each database search for publication year (2000-). Searches returned a total of 4,828 results.

APA PsycINFO (959)

((depression OR “depressive disorder*”) AND ((treatment N6 efficacy) OR (treatment N6 effective*) OR (intervention N6 efficacy) OR (intervention N6 effective*) OR "treatment outcome*") AND (diet OR food OR nutrition OR “dietary intake” OR “food intake” OR “nutrition intake” OR “diet therapy” OR "nutrition therapy"))

CINAHL (729)

((depression OR "depressive disorder*" OR (MH "Depression")) AND ((treatment N6 efficacy) OR (treatment N6 effective*) OR (intervention N6 efficacy) OR (intervention N6 effective*) OR (MH "Treatment Outcomes+")) AND (diet OR food OR nutrition OR “dietary Intake” OR “food Intake” OR “nutrition intake” OR (MH "Diet Therapy+")))

Cochrane Library (807)

((depression OR “depressive disorder*” OR (MH "Depression") OR (MH "Depressive Disorder+") OR (MH "Depressive Disorder, Major")) AND ((treatment NEAR efficacy) OR (treatment NEAR effective*) OR (intervention NEAR efficacy) OR (intervention NEAR effective*) OR (MH "Treatment Outcome+")) AND (diet OR food OR nutrition OR “dietary intake” OR “food intake” OR “nutrition intake” OR (MH "Diet Therapy+") OR (MH "Nutrition Therapy+")))

MEDLINE (1,522)

((depression OR “depressive disorder*” OR (MH "Depression") OR (MH "Depressive Disorder+") OR (MH "Depressive Disorder, Major")) AND ((treatment N6 efficacy) OR (treatment N6 effective*) OR (intervention N6 efficacy) OR (intervention N6 effective*) OR (MH "Treatment Outcome+")) AND (diet OR food OR nutrition OR “dietary intake” OR “food intake” OR “nutrition intake” OR (MH "Diet Therapy+") OR (MH "Nutrition Therapy+")))

Scopus (811)

TITLE-ABS ((depression OR “depressive disorder*”) AND ((treatment W/6 efficacy) OR (treatment W/6 effective*) OR (intervention W/6 efficacy) OR (intervention W/6 effective*) OR "treatment outcome*") AND (diet OR food OR nutrition OR “dietary intake” OR “food intake” OR “nutrition intake” OR “diet therapy” OR "nutrition therapy"))
